# “Walk me through the final day”: A thematic analysis study on the family caregiver experience of the Medical Assistance in Dying procedure day

**DOI:** 10.1177/02692163241248725

**Published:** 2024-05-09

**Authors:** Rinat Nissim, Paige Chu, Alison Stere, Eryn Tong, Ekaterina An, Debbie Selby, Sally Bean, Elie Isenberg-Grzeda, Gary Rodin, Madeline Li, Sarah Hales

**Affiliations:** 1 Department of Supportive Care, Princess Margaret Cancer Centre, University Health Network, Toronto, ON, Canada; 2 Department of Psychiatry, Temerty Faculty of Medicine, University of Toronto, Toronto, ON, Canada; 3 Sunnybrook Health Sciences Centre, Toronto, ON, Canada

**Keywords:** Medical Assistance in Dying, assisted dying, qualitative research, family, caregiver

## Abstract

**Background:**

Legalization of assisted dying is progressively expanding worldwide. In Canada, the Medical Assistance in Dying Act became law in 2016. As assisted dying regulations evolve worldwide, comprehending its subjective impact and broader consequences, especially on family members, becomes pivotal for shaping practice, policy, and training.

**Aim:**

The goal of this study is to understand the experience of family caregivers on the assisted dying procedure day.

**Design:**

Qualitative, thematic analysis, research using semi-structured interviews.

**Setting/participants:**

Family caregivers of patients who received assisted dying in two hospitals in Canada were recruited. Interviews were conducted at least 6 months after patient death. Conceptual saturation was achieved after analyzing 18 interviews.

**Results:**

While caregivers expressed gratitude for the availability of Medical Assistance in Dying, they also described the procedure day as potentially jarring and unsettling. We identified five aspects that shaped their experience: attuned support from the clinical team; preparation for clinical details; congruence between the setting and the importance of the event; active participation and ceremony; and pacing and timing of the procedure. Together, these aspects impacted the level of uneasiness felt by caregivers on the procedure day.

**Conclusions:**

This study emphasized the importance of a family-centered approach to delivering Medical Assistance in Dying. It underscored recognizing the needs of family caregivers during the procedure day and offering strategies to ease their experience. Healthcare providers in jurisdictions where assisted dying is legal or deliberated should consider the applicability of these findings to their unique context.

**What is already known about the topic?**

Current literature predominantly focuses on the moral permissibility and legislative frameworks of Medical Assistance in Dying.

The subjective experience and broader implications of Medical Assistance in Dying, including the impact on families, are still not well understood.

**What this paper adds?**

Through qualitative interviews with family caregivers of patients who died following Medical Assistance in Dying at one of two large urban hospitals, we found that while caregivers were grateful for the availability of assisted dying, they also experienced the procedure day as surreal and unsettling.

The study identified five key aspects that shaped the overall experience of family members on the procedure day: attuned support from the clinical team, comprehensive preparation for the clinical details of the day, alignment between the physical environment and the significance of the day, active participation and ceremony, and the pacing and timing of the procedure.

**Implications for practice, theory, or policy**

The study highlights the importance of a family-centered approach in the delivery of Medical Assistance in Dying, emphasizing the need to address the experiences and needs of family caregivers during the procedure day.

Our findings on the subjective experience of the procedure day can inform future policies, research and training initiatives, ultimately enhancing the delivery of Medical Assistance in Dying to meet the needs of all stakeholders involved.

## Introduction

Globally, the legalization of assisted dying is steadily gaining ground.^1,2^ As more jurisdictions worldwide introduce legislation for assisted dying, a variety of policies and procedures have emerged. In Canada, the Medical Assistance in Dying Act became law in 2016.^3^ This legislation allows assisted deaths for individuals aged 18 years and above facing imminent natural death and enduring unbearable physical or psychological distress, provided they are capable of giving consent. The Act also mandated a 10-day waiting period between approval and the procedure. However, this requirement was removed in 2021. While the Act permits both self-administered and clinician-administered procedures, the latter method remains predominant,^3^ as observed in other jurisdictions as well.^2^

When the 2016 legislation came into effect, Medical Assistance in Dying programs in Canada were established in an ad hoc manner with minimal guidance.^4-6^ However, Canadian research has since been pivotal in deepening our understanding of the subjective experience and broader implications of assisted deaths, including the impact on families. Current research, predominantly from European (particularly Swiss) and North American (specifically, Oregonian and Canadian) sources suggests that family members are typically involved in all stages of the assisted dying process^7-12^ and that their involvement with the assisted dying process may impact their emotional well-being and shape how their grief unfolds.^7-10,13-16^.

In a previous qualitative publication,^12^ we described the experience of Canadian family members of cancer patients who requested assisted death as a “race to the end,” with the ultimate goal of creating an ideal dying experience for the patient while balancing a threat to capacity that would undermine their access to the procedure. Our analysis also highlighted the unique stress they experience related to organizing and preparing for the procedure day. In the current manuscript, we aim to deepen our understanding of their experience during the day of the procedure itself. Notably, only one prior study has examined the experience of the procedure day, but from the perspective of healthcare providers working in hospitals or homecare settings in Belgium.^17^ Our study is focusing on the family members’ perspective, with the goal of highlighting their needs and shaping policies, clinical guidelines, and family-centered interventions for the procedure day.

## Methods

### Design

Data were generated through semi-structured interviews and analyzed using reflexive thematic analysis, informed by the methodology of methodical hermeneutics.^18,19^ This is a systematic approach to generating a new understanding of a phenomenon of interest. This understanding is developed recursively by making tentative interpretations regarding the meaning conveyed in interview transcripts and constantly checking and revising these interpretations against additional data.

### Setting

Recruitment took place within two large urban hospitals in Toronto, Ontario, Canada.

### Participants

Participants were recruited as part of an ongoing longitudinal, mixed-methods study of the patient and caregiver experience of Medical Assistance in Dying.^12,20^ Inclusion criteria involved adult family caregivers of patients who underwent assisted dying and had sufficient English fluency.

### Recruitment

Eligible caregivers were identified by the clinical team to the research coordinators. They were invited to participate in a semi-structured interview at least 6 months following patient death. All participants provided informed consent.

### Sample

Aligned with guidelines for purposive sampling,^18,19^ participants were selected based on attributes considered pertinent to the experience of the procedure day. For example, recognizing that different types of relatives may have distinct roles and emotional connections to the patient, which may impact their perceptions of the procedure day, we included individuals with various relationships (e.g. spouse and child). Additionally, in light of the impact of the COVID-19 visitor restrictions on attendance at the procedure day,^20^ we ensured equal sampling both before and during COVID-19 restrictions to address any potential variations in experiences arising from these circumstances.

### Data collection

Semi-structured interviews were conducted by EA and ET. Participants were interviewed by phone, video, or in-person at the participants’ discretion. The interview guide explored caregivers’ experiences encompassing assisted dying in general, the procedure day, bereavement, and their support needs. It was continuously refined in response to emerging themes. All interviews were audio-recorded, transcribed verbatim, and de-identified.

### Data analysis

Transcripts were managed using the NVivo12 software. Initial coding was conducted jointly by PC and AS, who met regularly to analyze interviews, discuss discrepancies, and create codes. Themes related to the experience of the procedure day were generated through comparative analysis of the initial codes and expanded upon through an iterative process of inductive coding to depict the caregiver experience during the day. The process was documented through memos and reviewed in weekly meetings with RN, SH, and ET. These weekly discussions enabled a shared contextual understanding and collaborative co-construction of data meaning, and helped facilitate reflexivity, mitigate biases and inform the selection of the next transcript to analyze. Transcript selection and analysis concluded upon our determination that the generated themes capture the most meaningful aspects of the data within the scope of the study.^19^

## Results

Transcripts of interviews with 18 caregivers, of 15 unique patients, were analyzed. All patients underwent assisted dying in a hospital setting through intravenous administration of lethal medications, between November 2018 and August 2021. The mean age of patients was 75 years, with a range of 49–95 years. Most patients (13/15, 87%) had a primary cancer diagnosis. Interviews ranged from 6 to 13 months post-death. The mean age of caregivers was 56 years, 50% were women and 55% were adult children of the patients (see Table 1 for additional characteristics of the sample). All were present during the procedure.

**Table 1.** Participants’ demographics (*n* = 18).

Characteristics	*N*	%
Age (years)	Mean (SD): 56.83 (14.48) Range 29–83	
Time since patient death at time of interview (months)	Mean (SD): 7.47 (2.15) Range: 6–13	
Gender		
Men	9/18	50
Women	9/18	50
Ethnicity (White)	18/18	100
Marital status		
Married/common-law	13/18	72.22
Separated/divorced	1/18	5.56
Single	1/18	5.56
Widowed	3/18	16.67
Religion		
Agnostic	3/18	16.67
Atheist	1/18	5.56
Christian	1/18	5.56
Jewish	4/18	22.22
None	5/18	27.78
Prefer not to answer	4/18	22.22
Highest level of education completed		
High school	6/18	33.33
College/trade	2/18	11.11
Undergraduate	5/18	27.78
Post-graduate/professional school	5/18	27.78
Combined family household income		
$30,000–$56,999	2/18	11.11
$60,000–$99,999	1/18	5.56
$100,000–$199,999	5/18	27.78
$200,000+	5/18	27.78
Prefer not to answer	5/18	27.78
Relationship with patient		
Child	10/18	55.56
Spouse	4/18	22.22
Other (parent, grandchild, and siblings’ children)	4/18	22.22
Time of procedure		
Pre-COVID-19 pandemic	9/18	50
During COVID-19 pandemic	9/18	50

### The experience of Medical Assistance in Dying procedure day from the family caregiver’s perspective

Participants expressed feelings of gratitude for the availability of assisted dying and spoke of the comfort and reassurance that it offered to them by honoring the patient’s autonomy and addressing their suffering. As one participant shared: “It was relief. It was happiness that my mom didn’t have to be in that pain anymore.” At the same time, the procedure day was a disorienting experience for families with participants describing the day as “the twilight zone” or “surreal”:

“If I had to pick one word to describe this whole thing, the whole thing? It’s surreal, not bad – it’s what my mother wanted, but it was kind of, like, ‘What just happened?’ It’s very strange.” [P.629-1].

Our analysis identified five inter-related aspects of the procedure day that shaped the overall experience and affected the level of comfort felt by caregivers during the procedure day: (1) attuned support from the clinical team; (2) preparation for the clinical details; (3) congruence between the setting and the importance of the event; (4) active participation and ceremony; and (5) pacing and timing of the procedure.

#### Attuned support from the clinical team

Participants repeatedly described the caring approach of the Medical Assistance in Dying team, which helped them throughout the procedure day. They shared that especially when the team was able to get to know the patient prior to the procedure day, they could provide care that was attuned to the specific preferences, wishes, values, and needs of the patient and their caregivers:

“She [Medical Assistance in Dying provider] would always take my opinion into account, which was amazing, she was really kind. . . she was compassionate. For her, this is another patient, but for us, this is our grandmother, and she was wonderful. She was so good. . .they [the Medical Assistance in Dying team] really put [our grandmother’s] feelings and thoughts at the forefront, like rather than imposing what they thought a regular patient might want in this situation. It was very much guided by her preferences and that was amazing.” [P.507-1]

The compassionate attitude of the clinical team was also beneficial in situations in which it was not possible to fulfill all of the patient’s wishes. For example, when the patient desired the procedure to be carried out at home:

“My mom wanted to be at home with family, whoever that was, it was just me or my sister or whatever, and yet we had total strangers in the room when this was happening, but it didn’t feel strange. That’s because of the types of people that they were. It was kind of calming in a sense too.” [P.516-1]

Lastly, participants appreciated the attentive support of the clinical team during the immediate aftermath of the patient’s death. This was a time when some participants felt particularly overwhelmed. As one participant shared: “At that point he was gone and we’re the ones that needed the support. Well, I did anyway because I no longer had to be the rock.” Participants expressed gratitude for not being “rushed out” and allowed time to process their emotions and for instances where a team member sat with them or engaged in conversation:

“And then, I don’t know if she was a doctor or a nurse, she was very helpful, she took off my mom’s jewelry and she gave it to me – ‘cause I asked her to. . . [cries] I sat there for a little bit. I was very glad that none of the — none of the doctors or nurses rushed me, which was very, very great. . .and afterwards I remember the doctor came in and said, ‘You did a great job’. Which was like, that’s really good to hear. It’s good to know that you didn’t let somebody down.” [P.638-2]

The importance of attunement to caregivers’ emotions immediately after the patient’s death was also demonstrated in situations where their emotional needs were not fully addressed, as illustrated in the quote below:

“That same day, within 15 minutes or so of the procedure, one of the nurses came in or the doctor came in and reminded us that this was a teaching hospital, and would I or would we be OK if a nurse came in to watch the procedure from a learning perspective. My mother supported that ‘cause she’s always been in support of that sort of thing. . .What I found though was that the doctor was in the room and then there were three, let’s call them observers? I didn’t like that. At the end of the day, as my mother was actually dying and then deceased. They were there and that – I felt because I was having a very emotional time at the time, that felt constrictive to me, to be going through that experience for me and to have onlookers. . .It felt like it needed to be a private moment and I didn’t get that.” [P. 620-1]

#### Preparation for the clinical details

The enormity of the procedure day meant that all details of that day, including those participants referred to as “minor,” were pronounced and significant. Participants spoke about the importance of a “talk ahead of time so you knew what was going to happen, you knew exactly how physically it would look and feel.” The degree of preparation for the clinical details, however “small” and “minor,” shaped caregivers’ sense of comfort during the procedure day. Participants who felt ill-prepared or taken aback by certain aspects of the intervention described those details as unexpectedly jarring, which left them feeling distracted and unsettled during the procedure. For example, some participants reported their difficulty and confusion regarding the need to reduce the patient’s pain medication on the day of the procedure to maintain capacity for consent. Many also described being “shocked” by the amount and size of procedure syringes:

“At the time of his death, all the procedure itself had been explained clearly and we all know what was happening. . .But it was the fact that the team came in with the drugs and the equipment and the syringes were visible. I think [the Medical Assistance in Dying patient] tried to make a joke when he saw that because that’s what he did all the time, especially if he was nervous. But I remember being quite shocked by the view of the big needles, it was just a kind of a distraction. I mean, we all knew what was happening, but when I saw those needles, that’s where both of us went to look right away. And it gave me a kind of queasy feeling, but he told the joke and I think we got over it, but I feel like maybe it’s a legal requirement that they be displayed? But I would say I’d rather not have that memory in my head. It comes up often in my memory and I would have preferred to focus on his face.” [P.612-1]

Conversely, other participants commented on appreciating when providers took the time to explain these clinical details, however “minor,” while administrating the procedure, and how this clear, ongoing, communication helped them feel more in control in this situation:

“I mentioned earlier that [the Medical Assistance in Dying provider], who was performing the procedure was very, very calm, very soothing in the way that she spoke. She explained how the process was going to work. As she started to initiate the process and my mother was falling asleep, she was explaining to me each of the needles or each of the syringes that she was attaching to the system, what they did and how what was happening to mom at that stage. All very, very helpful for keeping your head on straight [chuckles]. So that aspect of it, I can’t imagine how it could have gone any better.” [P.620-1]

#### Congruence between the setting and the importance of the event

For many participants, there was a mismatch between the impersonal nature of the hospital setting and the importance of their loved one’s last moments. Some participants described feelings of guilt or regret about the hospital room where the assisted death occurred. Several expressed a wish for an improved physical environment, and for a dedicated, less “clinical” room in the hospital that they could decorate and personalize and that would be more “sensitive” to and “congruent” with the intensely personal experience of assisted dying:

“The facility itself, i.e. the room itself and the wing, the more I reflect on it, I would quite candidly. . .I would say to the hospital, invest some money and have one or two dedicated rooms that are not so clinical. So it’s not that [the Medical Assistance in Dying patient] didn’t have a private room or anything like that but I sort of sit back and it’s like an overly sterile hospital room where I think for someone who’s ending their life, not that it’s going to be like walking into a hotel room, but I would, that would be the one thing that I would say that they should really go back and re-assess, is thinking about the family, the use of the room and so on. . .that would be one of the strongest things that I would say is, I think you’d need to think about the setting because the work that the team does, it not congruent with the setting, the feeling that people have at the end. And then I think about. . .what if the room was a little bit bigger or what if there were couches and chairs but not just like plastic folding chairs, what if there was like proper seating. . .so that people can be comfortable knowing that this is their last two hours with [the person].” [P.542-1].

Participants described trying to make the best of the hospital setting and creating the most comfortable and supportive environment for the patient. As discussed earlier, the enormity of the procedure day meant that they paid attention to even “tiny” details about the setting:

“I was trying to plan which side his port for the drug would be, on the left side of the right, and I picked the side that would be furthest away from the window so that the medical team could be on that side of the bed, and then I could be in his view of the window. I know this sounds- [laughs] crazy! But, I picked the PICC line location based on the ward room he was in palliative care, but then the next morning they transferred him to a private room and so it was kind of reversed, which is a very tiny detail. But I kind of in that circumstance, I suppose I wanted to have a bit of control of scenery. . . I just wanted his view to be a certain direction, I suppose.” [P. 612-1]

#### Active participation and ceremony

The ability to be involved in the planning of the assisted death day provided a sense of meaning and control for caregivers during an emotionally tumultuous time. With assisted dying being a novel experience, without established customs and rituals, some caregivers expressed a sense of uncertainty about how to participate effectively on the day itself and provide the best possible experience for the patient. Some caregivers described self-doubt during the bereavement phase, questioning if they could have done more to orchestrate a better experience for their loved one, and wishing for resources beforehand to learn how they could have expressed their love and care on the procedure day and bid a meaningful goodbye:

“That was the part I didn’t really have guidance on but that’s okay because, maybe that was just something that every family figures out for themselves, what they’re going to do, ‘cause I understand there’s different things that go on in these things and maybe, should I have done something more in the room? I don’t know. You know, you second guess yourself and, you know, was it mostly just important for us to be there? And I read articles later that people had music and I never thought about stuff like that.” [P.648-1]

In contrast, some participants shared stories of ceremonies and “celebrations” with poems, music, candles, food, and drinks that were organized in advance and provided a sense of structure and communal participation on the day. As the quote below illustrates, the ability to actively participate and ritualize the day brought a sense of positive ambience, joy, and connection:

“The room was packed. We had brandy, we had a playlist, my daughter had come in the day before and drawn pictures, we had them stuck up on the wall with medical tape, it wasn’t a sad–it didn’t feel somber, everyone was laughing right ‘til that last second, and then it was like it had kind of changed, like oh someone died, but until then it felt upbeat, and we all wore her favourite color, turquoise, so we all wore something that had turquoise in it. We all matched, which was cute. It felt nice, and she knew exactly what was happening ‘cause there were a few things that I said in that last week where her strength waned, and they realized she needed a bunch of infusions, and she got it, and like oh my gosh, she was so coherent, she was so happy, she was so clear, she was so sure of herself. And seeing that in the person that is about to go through this. . .it’s hard to feel sad when they’re not sad at all. She wasn’t. We did some videos for her from around the world, people were calling in, so there was a lot of people, even people beyond who were in that room who were very much involved.” [P.507-1]

#### Pacing and timing

Some participants reported experiencing consternation and unease related to the timing and pacing of the procedure. For some, this was due to the procedure being delayed or uncertainty about when it would begin and how long it would take. However, for most, the cause of consternation was the unexpectedly quick pace of the procedure. They felt unprepared for the speed at which their loved one’s death occurred, causing them to feel overwhelmed and concerned about whether the patient was ready for such a sudden death, and wished they had received more information or prompts to help them keep up with the speed of the procedure:

“I think I would have liked to have known, ‘Ok he’s gonna go to her one arm and inject something and then he’ll go into her other arm and inject something.’ And again, honestly, maybe they did tell me that, I don’t recall in the moment. And then it seemed all of the sudden that he’s injecting her [laughs] it’s like, ‘Ah wait!’ And we were holding her hands and then it just seemed to be over really fast, which, I mean, it needed to be. But again, if I could go back again and wave a magic wand and change something, maybe a little prompt from him or the nurse like, ’Do you wanna say anything else to your mom right now?’ We said something quickly but almost I wish for another couple of minutes in that moment.” [P.629-2]

## Discussion

This study contributes to the growing understanding of how assisted dying affects the broader family unit,^7 [Bibr bibr8-02692163241248725] [Bibr bibr9-02692163241248725] [Bibr bibr10-02692163241248725] [Bibr bibr11-02692163241248725] [Bibr bibr12-02692163241248725] [Bibr bibr13-02692163241248725] [Bibr bibr14-02692163241248725] [Bibr bibr15-02692163241248725] 16^ with its unique focus on the experience of family caregivers during the procedure day. The 18 caregivers interviewed expressed appreciation for the option of assisted dying, as it provided a way for patients to exercise their autonomy and alleviate their suffering. They also shared that the procedure day was a surreal and potentially unsettling experience for them. The study identified five inter-related aspects that contributed to the overall experience of family members and affected the level of comfort they felt during the procedure day: attuned support from the clinical team; preparation for clinical details; congruence between the setting and the importance of the event; active participation and ceremony; and pacing and timing of the procedure.

In a recent qualitative study conducted in Belgium,^17^ which explored the perspectives of providers on their experience of the assisted dying procedure day, providers described aiming to position themselves inconspicuously, so as not to disrupt the intimate and emotional moments between families and their loved one. However, our findings suggest that families may rely on the clinical team throughout the procedure day for guidance and support, rather than wanting them to remain in the background, and that the compassionate and supportive demeanor of the clinical team, along with their guidance during and immediately after the procedure, helped caregivers feel less disorientated and overwhelmed.

A common unmet need among family members during a patient’s last month of life is understanding what to expect during the time of death.^21^ Our findings emphasize this unmet need in the context of the procedure day and highlight the role of providers in preparing family members, to the extent possible, for the procedure day, and offering explanations throughout its entirety. When details of the procedure, such as the quantity and size of procedure syringes, were unexpected, they had a jarring effect on caregivers. Adequate preparation, before and throughout the procedure, including attention to seemingly minor details, helped caregivers maintain focus on the patient and effectively manage their own emotional reactions. Additionally, distress arose when there were delays, a hurried pace, or unclear communication about the start and duration of the procedure. These findings echo previous qualitative studies that highlight the negative impact of delays or rescheduling of the procedure^22^ or of other deviations from what was expected.^9^ Providers^17^ also described their own distress when they were delayed or unable to carry out all the arrangements that they had made with the patients and their families, with last-minute changes perceived as interfering with their ultimate goal “to achieve a serene transition from life to death” (p. 6).

Our analysis suggests that there is a fundamental contrast between the clinical and sterile hospital environment and the deeply personal experience of losing a loved one. This aligns with previous research about the importance of the setting in end-of-life care.^23–25^ While private residences are the primary setting for assisted deaths,^9^ many patients in acute care are too unwell to be transferred home before the procedure and some prefer the hospital setting to shield their families.^26^ In such cases, efforts to mitigate the impersonal nature of the hospital and create an environment that respects the individual’s preferences and fosters a homely and serene atmosphere during the procedure may be helpful. For instance, providing patients and families with the opportunity to decorate the room with personal items, bringing in favorite blankets or photographs, can contribute to a more intimate setting. While efforts to improve hospital environments can enhance the experience of all hospital deaths, our paper uniquely highlights the unsettling dissonance between caregivers’ envisioned procedure day and the stark reality of the hospital room, particularly given their aspiration to orchestrate an ideal death experience.^12^

Lastly, our study identifies the benefits of incorporating ceremonial activities during the procedure day. Similarly, Beuthin et al.^13^ described how knowing the exact death date activated families of patients requesting assisted dying to organize both formal and informal ceremonies during various stages, including on the procedure day itself, and that these ceremonies were a crucial aspect of the grieving process. Deathbed rituals were an important part of dying in pre-modern societies, forming the basis for bereavement.^27,28^ The secularization and individualization of modern societies and the medicalization of death led to a decline in the practice of such rituals accompanying death,^28-31^ with assisted dying being perhaps the most secular and individualized form of death.^32,33^ Further research is needed to explore ways to integrate rituals and ceremonies during the procedure day, as well as developing adequate resources for families who wish to incorporate them.

Although assisted dying is characterized as a patient-focused medical procedure that emphasizes patient’s autonomy and personal control,^1^ our findings underscore the importance of a family-centered approach when delivering assisted dying. Interestingly, our participants often dismissed aspects of the procedure day that were distressing to them, labeling them as minor or insignificant, as they may have pre-emptively assumed that their emotions are less important than those of the patient. Although this self-silencing tendency is common among caregivers,^34^ ultimately, a good assisted death should include attention to the family’s capacity to create positive memories and to manage their grief.^15,17^

While assisted dying is often seen as incompatible with palliative care,^1^ studies with palliative care providers who were involved in assisted dying provision demonstrate that they perceived their involvement as congruent with their dedication to palliative care values, including the relief of suffering, practicing holistic and non-judgmental care, advocating for patient autonomy, and facilitating a good death, as defined by the patient.^35-38^ Our findings echo these studies, underscoring the importance of incorporating palliative care expertise and values in assisted dying provision, particularly in offering family-centered care on the day of the procedure. The legalization of assisted dying in various jurisdictions has not been accompanied by sufficient guidance in clinical practice. Current educational frameworks focus on discussing assisted dying requests and assessing eligibility.^39^ By incorporating our findings and insights from other qualitative studies on the subjective experience of assisted death and its aftermath^7,9^ we can enhance training initiatives and bridge the existing educational gaps in delivering assisted dying, ultimately increasing competency in delivering assisted dying that encompasses a comprehensive consideration of the needs of all stakeholders, including family caregivers.

### Limitations

There are several limitations to our study. Our findings may be influenced by individuals’ current bereavement-related distress and memory bias^40^ as well as selection bias, as individuals who had the most complicated and distressing experiences or who were fundamentally opposed to assisted dying may not have been willing to participate. Although consistent with the demographics of assisted dying recipients in Ontario,^3,4^ our sample is comprised of white individuals with a higher socio-economic status, and our results may not be applicable to caregivers from marginalized groups who may view assisted dying through a different cultural or socioeconomic lens. Our study is also limited to hospitals and additional research is needed to explore the experience of assisted dying in home and community settings, where different aspects may influence the procedure day experience. Importantly, half of our sample described procedures occurring during the COVID-19 pandemic. The COVID-19 pandemic and associated public health measures have significantly altered end-of-life experiences, especially those occurring in hospitals, including those related to assisted deaths.^20,41,42^ We therefore purposefully analyzed both pre- and post-pandemic experiences and concluded that while the pandemic have magnified certain distressing aspects of the procedure day, these aspects already existed before the pandemic. However, the pandemic might explain why our study did not find improvements in practice over the study period.

The landscape of assisted dying legislation and procedures is in a state of continuous evolution. In jurisdictions where assisted dying is permissible or being deliberated, healthcare providers, educators, and policymakers should assess the applicability of our findings within their own context. Our findings are particularly pertinent to clinician-administered assisted dying, which remains the prevailing practice across all jurisdictions.^2^ With the unfolding progression of legislation in Canada and other jurisdictions, it is essential to involve caregivers as key stakeholders in the process, influencing approaches to their support.

## Conclusions

The current study adds valuable insights to the existing literature on assisted dying. The findings suggest that fostering compassionate care, promoting thorough preparation that encompasses all procedural details, providing a setting that aligns with the intensely personal nature of the assisted dying experience and incorporating opportunities for ceremonial activities can enhance the overall experience of the procedure day for family caregivers as well as their recollection and emotional response to the assisted death, even months later.
